# The role of cations in the interactions between anionic N-heterocycles and SO_2_

**DOI:** 10.1038/s41598-018-25432-6

**Published:** 2018-05-08

**Authors:** Chenchen Li, Dongmei Lu, Chao Wu

**Affiliations:** 1 Frontier Institute of Science and Technology, Xi’an Jiaotong University, Xi’an, Shaanxi 710054 China; 2 Department of Applied Chemistry, School of Science, Xi’an Jiaotong University, Xi’an, Shaanxi 710049 China

## Abstract

Our study shows that cation plays a more important role in the interactions between anionic N-heterocycles and SO_2_ than in the NHC-CO_2_ case. The adducts of NHC, SO_2_ and cation often exhibit multiple stable configurations with close energies rather than the only reported “CO_2_-sandwiched” planar NHC-CO_2_-cation structure. The structural diversity makes the models omitting cation inappropriate for predicting the SO_2_ capture products, which also leads to less clear trends of the cation effects than those observed in the CO_2_ case. The detailed cation effects are discussed in the text.

## Introduction

As a key air pollutant, SO_2_ poses great danger to the environment. Traditional desulfurization scrubs flue gas with sorbent agents like limestone or lime, however this technique is hampered by large amounts of useless or low-value by-products (e.g. calcium sulphite or sulfate). Moreover, once consumed, the sorbents cannot be regenerated as the by-products are too stable^1–4^.

Anionic nitrogen-containing heterocycles (NHCs) can serve as Lewis bases. Through the electron lone pair of the N atom, NHCs can form dative bonds with acid gas like SO_2_ and CO_2_. The adducts are far less stable than sulfites and sulfates, and they can be converted back into reactants simply by heating and/or vacuuming. Thus, NHC-containing ionic liquids (ILs) and metal-organic frameworks (MOFs) have been widely studied for reversible acid gas capture^5–13^. NHCs carrying negative charge are believed to dominate the interaction with acid gas, while the positively charged parts (cations) are often considered as charge balancing agents only^5,14–16^.

However, recent studies of the cation effects on the interactions between Lewis base and CO_2_ have displayed a different picture. In 2014, He and Dai groups reported that the introduction of lithium or potassium into organic bases improved their CO_2_ uptake to nearly equal molar level, a result of forming stable zwitterionic metal carbamate adducts with CO_2_ being sandwiched in the middle^17,18^. In parallel, theoretical works by Neaton group proved that the interactions between NHCs and CO_2_ were strengthened by the positively charged metal centers in NHC-grafted MOFs^19,20^. This was echoed by the experimental work of Long group in their diamine-appended MOFs, where the divalent cations (Mg, Mn, Fe, Co, Zn) were utilized to achieve high CO_2_ uptake by allowing CO_2_ to insert into metal-amine bonds^21^. Meanwhile, our group systematically studied the cation effects on the interactions between a group of conjugated NHCs and CO_2_, which revealed that the NHC-CO_2_-cation in-plane configuration was the most stable and especially the bivalent cations, due to their strong electrostatic interactions, significantly enhanced the binding with CO_2_^22^.

Since the two acid gases, CO_2_ and SO_2_, have a number of similarities in structure and property, we naturally wonder whether the interactions between SO_2_ and NHC-cation systems simply follow the same story line? Nevertheless, in a recent simulation research, Ai group reported that for alkyl phosphonium tetrazolide-based ([P_nnnm_][Tetz]) ILs, compared with the binding between the anion ([Tetz]^−^) and SO_2_, the binding strength of the anion-cation pair with SO_2_ decreased sharply by about 58 kJ mol^−1^ and resulted in reduced SO_2_ absorption capacity^23^, which was also observed in the studies of Wang group and Wang group using different ILs^8,24^. In their calculations, SO_2_ did not insert in between the anion and cation pair like CO_2_ did. Actually, SO_2_ rarely breaks the ion pair^8,15,23–27^. Still for simplicity, calculations of SO_2_ interacting with ILs were routinely carried out in the absence of cation since the cation effects are assumed to be negligible^5,7,9,10,12,28^. To date, whether cation promotes or restrains the binding between NHC and SO_2_ has not yet been clarified. Additionally, there are also evident differences in the geometric and electronic structures between SO_2_ and CO_2_, such as bond angle (118.7° for SO_2_ vs. 180.0° for CO_2_) and polarity (dipole 2.03 Debye for SO_2_ vs. 0.00 for CO_2_). How these differences are reflected in the interactions between the two gases (SO_2_ and CO_2_) and NHC-cation complexes calls for a systematic study.

**Figure 1:**
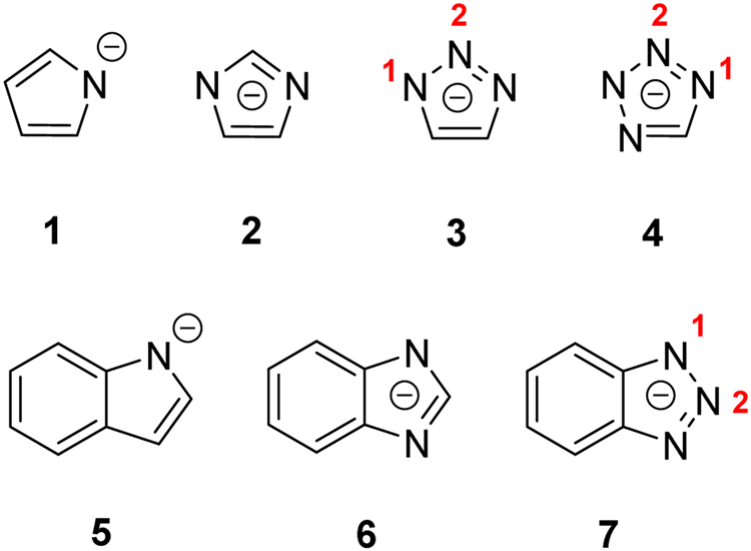
NHCs studied in this work. Multiple distinct SO_2_ interaction positions are labelled by numbers in red.

Here, we tried to elucidate the cation effects on the interactions between NHCs and SO_2_ by employing the density functional theory (DFT)-based calculations. In order to avoid the interference of complicated steric effects often found in ILs and to focus on the nature of the interactions, our systems were designed to consist of a number of representative conjugated anionic NHCs (Fig. 1) and simple cations (alkali and alkaline earth metals). We first explored the reaction potential energy surface (PES) of pyrrolide, one of the simplest anionic NHCs, with SO_2_ and Li^+^. From the PES, we identified multiple stable configurations of the pyrrolide-Li^+^-SO_2_ complex with nearly equal energies. After checking other NHC-cation-SO_2_ complexes, we found that multiple stable configurations are a common feature but the energy disparity among configurations is highly dependent on both the cation and anionic NHC. Due to the structural diversity of the complexes, we discovered that the lone pair orbital energy of NHCs is no longer a good descriptor to quantify their interactions with cation and SO_2_, unlike in the CO_2_ case. Finally, we concluded aspects about the cation effects and their influence on designing NHC-based ILs and porous materials for SO_2_ capture.

## Results and Discussion

We chose a series of commonly seen conjugated NHC anions (single-ringed structures pyrrolide **1**, imidazolide **2**, 1,2,3-triazolide **3** and tetrazolide **4** *and* double-ringed structures indolide **5**, benzene imidazolide **6** and benzene 1,2,3-triazolide **7**). We studied their interactions with SO_2_ and simple monovalent (Li^+^, Na^+^, K^+^, Rb^+^, Cs^+^) and bivalent cations (Be^2+^, Mg^2+^, Ca^2+^, Sr^2+^).

### SO_2_ absorption and desorption

**Figure 2:**
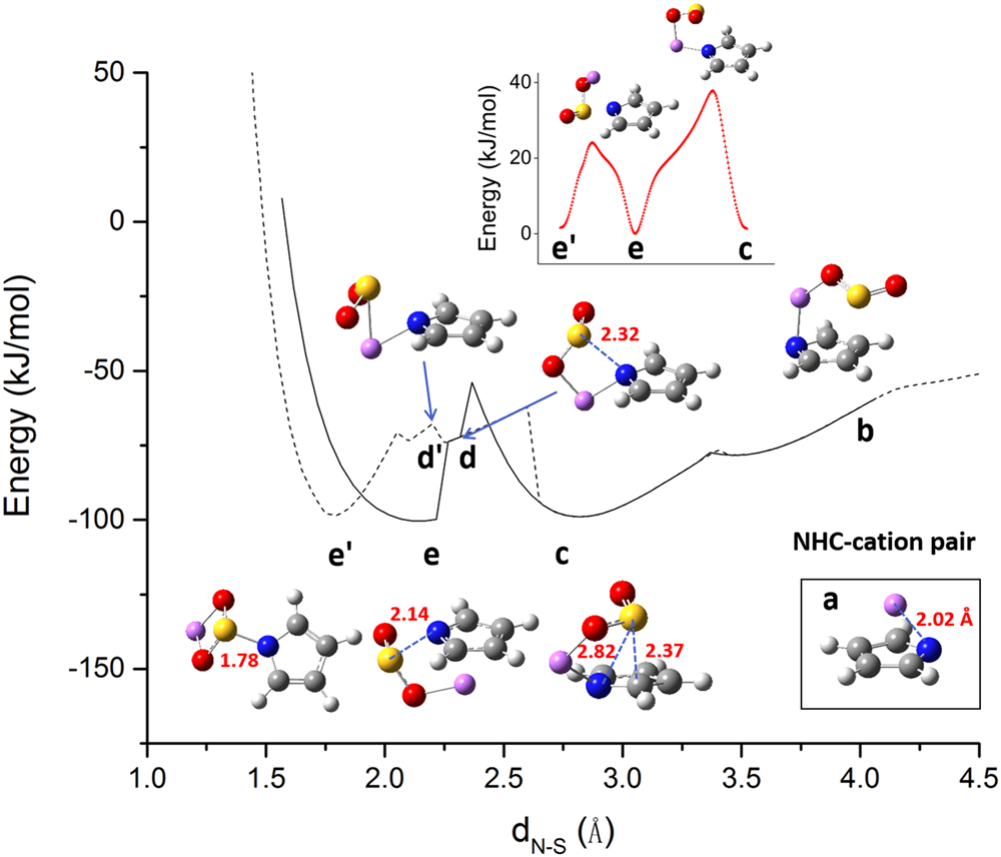
PES of Li^+^-assisted reaction between pyrrolide **1** and SO_2_. Solid line stands for the absorption process and dotted line stands for the desorption process. Key structures on the PES’ are labelled and plotted. The energy reference point is chosen when the (NHC-Li^+^) complex and SO_2_ are at an infinite distance. Bottom right inset: the **1**-Li^+^ complex. Top inset: transition energetics and transition states from **e′** to **e** and from **e** to **c**, respectively.

The reaction was explored by scanning the distance between the N atom (NHC) and the S atom (SO_2_), which is an efficient and approximate way to explore the reaction energetics and identify key intermediates. The scans were done in two ways. The first started with SO_2_ being far away (> 4.5 Å) from the NHC-cation complex and the N-S distance was gradually shortened until 1.5 Å. The second started with a NHC-SO_2_-cation complex analogous to the NHC-CO_2_-cation stable configuration (details see structure **e′** in Fig. 2) and the N-S distance was gradually elongated to over 4.5 Å. The scans were supposed to give information about absorption and desorption of SO_2_, respectively.

Li^+^ and pyrrolide **1** were used as the NHC-cation pair to help illustrate the processes (Fig. 2). The two scans of SO_2_ resulted into two distinct energy profiles. When SO_2_ is far away from **1** and Li^+^, the NHC and cation form an adduct **a** (Fig. 2a) featuring a (*η*^5^-NHC)Li type of binding (the binding energy is −682.2 kJ mol^−1^). The structure clearly differs from the planar complex composed of neutral NHC and Li^+^ reported in our previous work^22^ (their interconversions were described in details in ESI Fig. S1).

As SO_2_ approaches the complex, it pushes the Li atom over the N atom with one O atom and the Li-N bond is nearly perpendicular to the ring plane (Φ_LiNCH_ = −94.9°, Fig. 2b). Energy of the system keeps dropping until the N-S distance shrinks to 2.82 Å, where a stable structure **c** (Fig. 2c) is found. In structure **c**, the Li atom moves towards the ring plane (Φ_LiNCH_ = −40.8°) and the O-Li-N interaction (bond lengths are 1.83 Å and 1.93 Å, respectively) drags SO_2_ closer to the ring plane (the shortest S-C distance is 2.37 Å). Through a couple of key intermediates at the N-S distance of around 2.7–2.2 Å (Fig. 2d), SO_2_ moves beyond the far end of the N atom in the ring but still stays above the ring plane. Then the S atom only interacts with the N atom rather than with the pyrrole ring as in previous configurations. The Li atom is pushed aside but the O-Li-N interaction remains (bond lengths are 1.83 Å and 1.95 Å, respectively). When the N-S distance is 2.14 Å, intermediate structure **d** (Fig. 2d) relaxes into a similar but more stable configuration **e** (Fig. 2e). Further decrease of the N-S distance leads to dramatic energy rise and no more stable configuration appears. Thus the whole absorption scan generates two stable structures (Fig. 2c,e) and does not produce the configuration with SO_2_ inserted in between the N(NHC)-Li bond like in the CO_2_ case^22^.

Nevertheless, this may be an artifact of constrained optimization (*i*.*e*. scans). The expected configuration **e′** (Fig. 2e′) with SO_2_ bidentating to the Li^+^ atom through its two O atoms was set as the initial guess structure for the desorption scan. Indeed it is stable at the N-S distance of 1.78 Å. Unlike CO_2_, SO_2_ and the pyrrole ring are not coplanar and both the O atoms stick out of the ring plane. Thus, Li at one terminal of the complex is out of the pyrrole ring plane as well. Furthermore, a smaller N-S distance leads to substantial energy rise (Fig. 2 dotted line). Then, from configuration **e′**, the desorption scan (elongation of the N-S distance) was carried out. As the N-S distances increases, Li^+^ moves towards N of NHC and SO_2_ detaches from the ring. The complex position switching process is best represented by an intermediate structure **d′** (Fig. 2d′) which features the ring-leaving SO_2_ above the ring-approaching Li atom. These position changes again results into the stable structure **c** at the N-S distance of 2.82 Å. After that, the desorption and absorption energetics overlap.

From the two scans, three nearly degenerate (ΔE < 2 kJ mol^−1^) stable structures **c**, **e** and **e′** are identified *and* all exhibit strong interaction with SO_2_ (binding energy of about −140 kJ mol^−1^). The great structural variation in the three configurations reflect complex NHC-cation-SO_2_ interactions. Transformations between either two configurations were achieved through the IRC method and are found to require a relatively low barrier (< 40 kJ mol^−1^, transition states shown as the top inset in Fig. 2), indicating that the SO_2_ absorption product is multimorphic and flexible to interconvert among different configurations. Still, **c** may be the most populated structure as it is the first stable configuration formed during absorption. This supports the observation that SO_2_ does not break the anion-cation pair^8,15,23–27^. More importantly, it shows that *both* cation and anion (NHC) interact with SO_2_ considerably, as the Li atom is dragged out of the pyrrole plane and the shortest S-C distance is only 2.37 Å.

In comparison, only planar structures with CO_2_ sandwiched in the middle of the NHC-cation pair have been reported^22^. After we scanned the reaction between pyrrolide **1** and CO_2_ in the presence of Li^+^, two metastable structures were obtained (ESI Fig. S2), which can be converted into the well-known planar product NHC-CO_2_-Li exothermically by 75.2 kJ mol^−1^ after overcoming a small (less than 10 kJ mol^−1^) energy barrier. It demonstrates that, compared with SO_2_, the multiple stable configurations of NHC-CO_2_-Li cannot coexist since the in-plane CO_2_-sandwiched configurations are much more stable and other less stable configurations can easily transform into it^22,29^.

**Figure 3:**
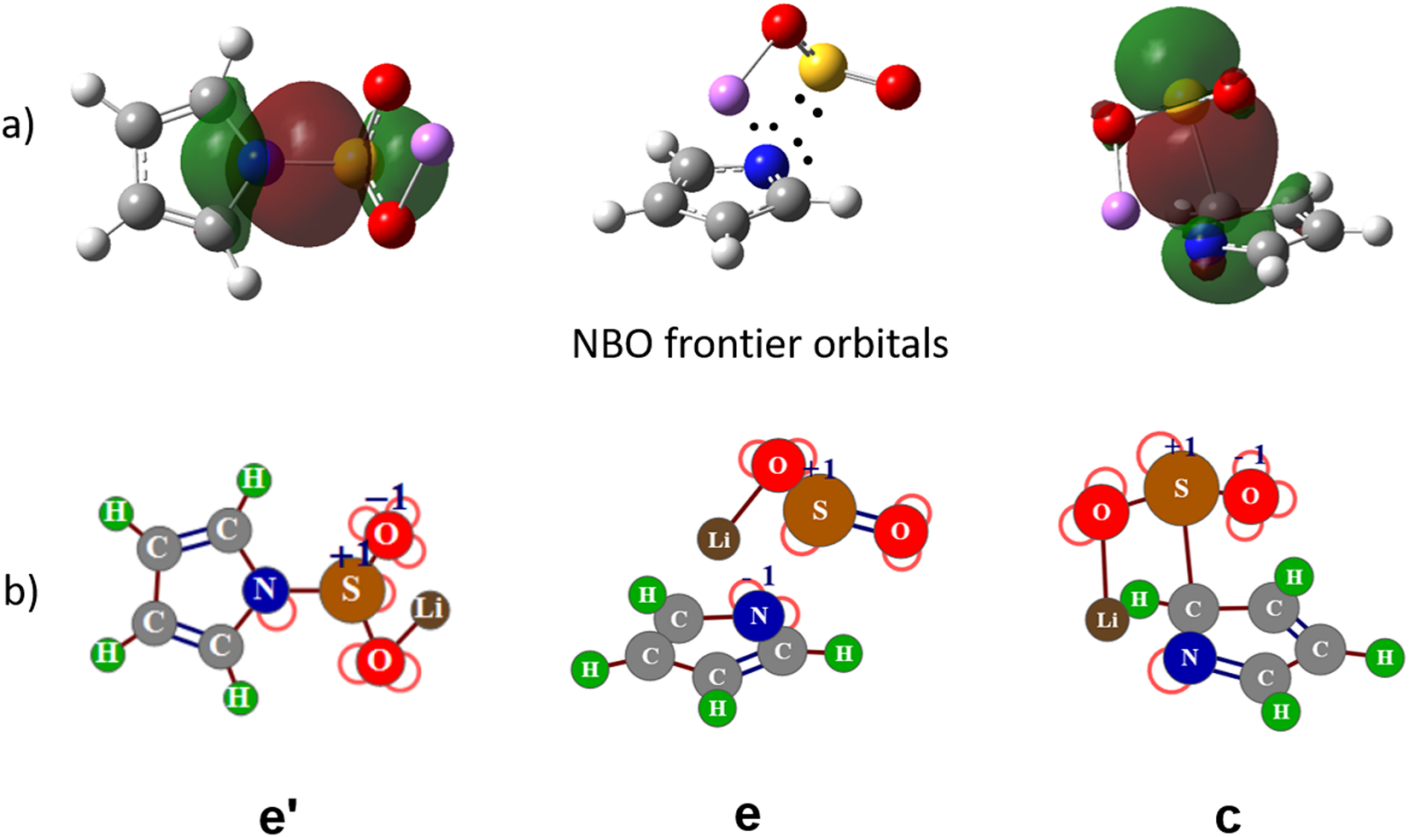
(**a**) NBO decomposition of the N/C-S bond of three stable configurations of pyrrolide 1-Li^+^-SO_2_, respectively. In **e**, its frontier NBO orbitals are represented by three electron long pairs on N (two pairs) and S (one pair). Dots represent electron lone pairs. (**b**) Leading Lewis resonance structures of the corresponding complexes. Red half circles represent electron lone pairs.

Furthermore, NBO and NRT analyses of the pyrrolide-Li^+^-SO_2_ complex were carried out to explore the nature of the interactions. The leading NRT structures are displayed in Fig. 3b and all major Lewis resonance structures and their abundances are shown in Fig. S3 (see ESI). For structure **e′**, like the interactions between anionic NHCs and CO_2_ in the presence of Li^+^^22^, pyrrolide donates its lone pair to SO_2_ to form a N-S *σ* single bond (Fig. 3a). The similar chemical bonding occurs in the case of structure **c**, whose *σ* bond is between the C and S atoms, while there is no S-pyrrole covalent bonding in the case of structure **e**. Thus in **e**, three electron lone pairs with two on N and one on S constitute the frontier NBO orbitals. Further bond order analyses confirmed that there is just a N/C-S *σ* single bond (NBO bond orders are 0.86 and 0.32 for **e′** and **c**, respectively), rather than the *π* bond observed in the CO_2_ case^22^.

**Figure 4:**
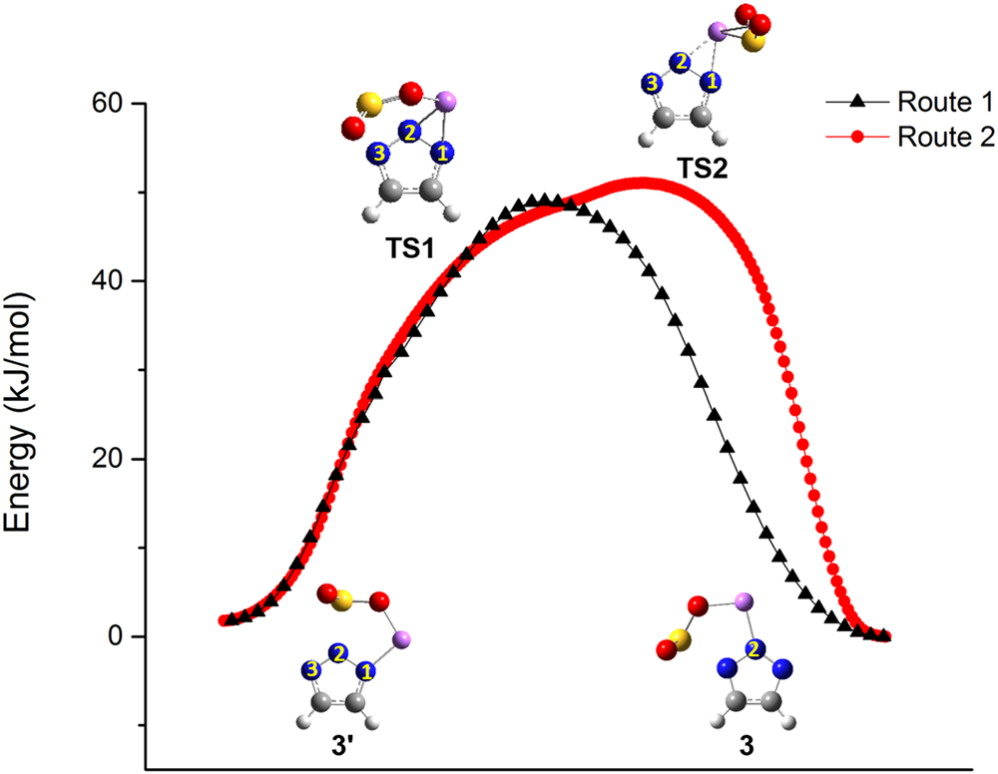
Two stable configurations of the **3**-Li^+^-SO_2_ complex and their interconversion implemented by the IRC method. N1 and N3 are not labelled in **3** as the two pathways lead to products with SO_2_ binding at either N1 or N3, but their structures are identical due to the same functional groups on the two C atoms.

For multiple-site anions, e.g. 1,2,3-triazolide (**3**), SO_2_ can interact with two unequal N sites (labelled as 1 and 2 in Fig. 1) to produce two stable structures in the presence of Li^+^ (ESI Fig. S4b). Interestingly, these two absorption products can also interconvert to each other through a moderate barrier (about 50 kJ mol^−1^) *via* two pathways, whose differences only lie in the way of SO_2_’s movement, while Li always bidentately binds to two N atoms (Fig. 4). Route 1 is called migration, as the Li atom in **3′** moves from N1 to N2 while SO_2_ is gradually pushed from N2 to N3. Route 2 is called swapping, as Li and SO_2_ switch their positions. During the transition, the Li atom stays in the NHC plane (Φ_LiNNN_ = 175.2°), while SO_2_ moves from N2 to N1 by going over one side of the NHC plane. It is noteworthy that although the same structure **3** is produced by these two different pathways, SO_2_ actually interacts with different N atoms (N3 in route 1 and N1 in route 2). Different substitution groups on the two carbon atoms should be able to make the pathways more distinct.

From the scan calculations of other NHC-Li^+^-SO_2_ systems, it can be clearly seen that multiple stable configurations are a common feature (ESI Fig. S4). Yet the energy disparity among the configurations may vary substantially. To get a more complete picture of the relative stability of the configurations, other variables such as the cation species including its charge need to be considered. Moreover, to make sure there was no significant stable structures omitted by the constrained scan calculations, we performed configuration search utilizing a Monte-Carlo method implemented in the Molclus program^30^.

### Stable configurations

**Figure 5:**
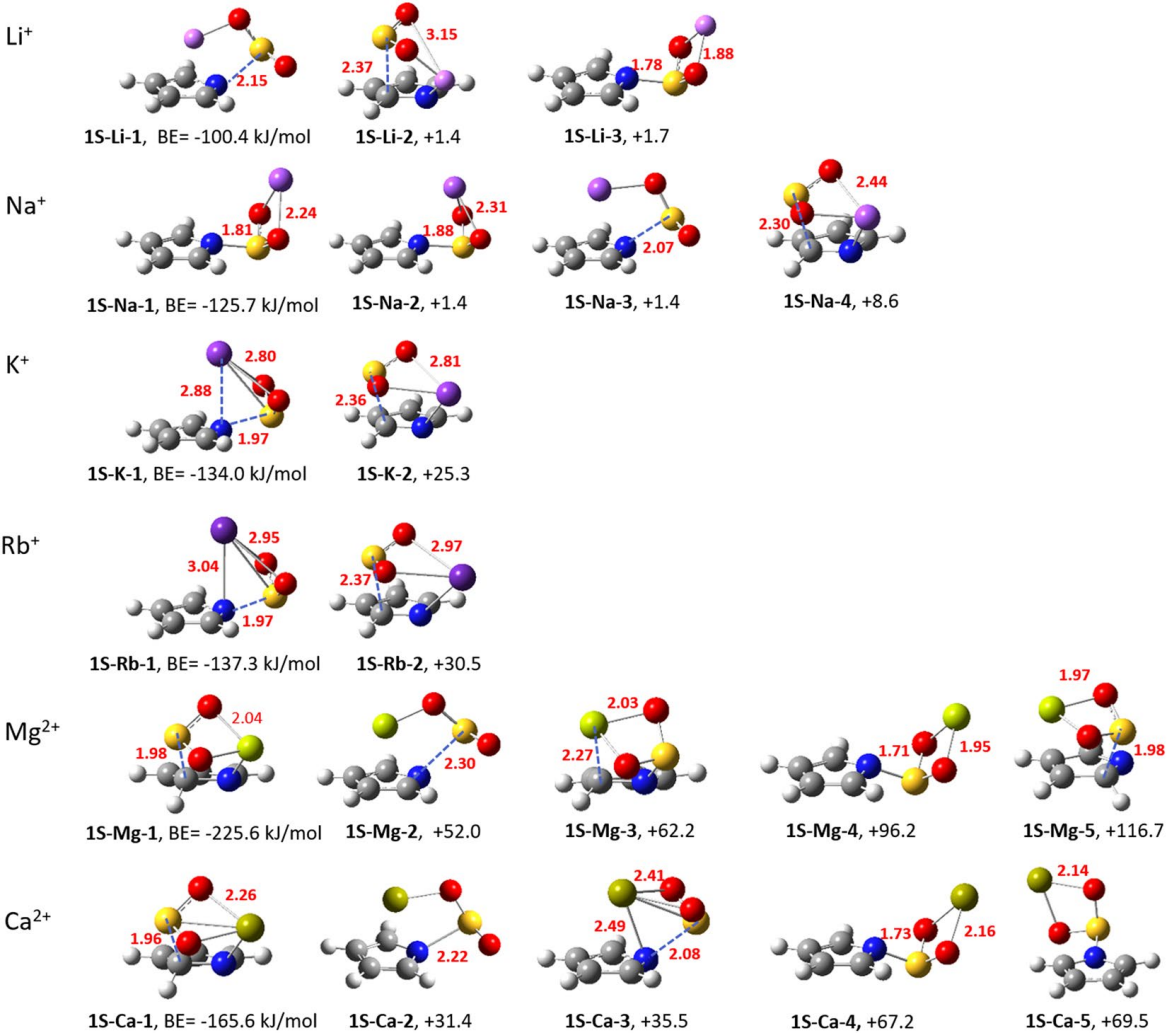
Stable configurations of **1**-cation-SO_2_ complexes. The name and the binding energy or the relative binding energy with respect to the most stable one (in kJ mol^−1^) are given below each configuration. Solid and dashed lines indicate the key bonds and distances of interest (in Å), respectively. Red is oxygen, blue is nitrogen, yellow is sulfur, gray is carbon, white is hydrogen, four light to dark purples are monovalent cations (Li^+^, Na^+^, K^+^ and Rb^+^) and two yellow-green are divalent cations (Mg^2+^ and Ca^2+^).

Again, pyrrolide **1** was taken as an example to illustrate the factors influencing the relative stability of multiple stable configurations. The three leading stable configurations of the **1**-Li^+^-SO_2_ system are relabelled as **1S-Li-1** (**e**), **1S-Li-2** (**e′**) and **1S-Li-3** (**c**) in the top panel of Fig. 5, which have all been obtained by the scans. In contrast, for the **1**-cation-CO_2_ systems (ESI Fig. S5a), the classical CO_2_-sandwiched in-plane structure is always the most stable configuration and the disparity in energy among configurations are much bigger than that in the **1**-cation-SO_2_ systems.

For the **1**-Na^+^-SO_2_ system, four states with close energies and substantial structural variations have been identified. The first configuration (**1S-Na-1**) is the most stable one and is similar to **1S-Li-3**, featuring the “expected” SO_2_**-**sandwiched structure. Only the second structure (**1S-Na-2**) has no close resemblance in the **1**-Li^+^-SO_2 _system and it differs from the first one by bending the Na end over SO_2_, approaching the pyrrole ring. However, for bigger alkali metals like K^+^ and Rb^+^, the energy disparity among configurations rises (over 25 kJ mol^−1^) quickly. The most stable configurations both mimic **1S-Na-2** but with the cation even closer to the ring, which reflects the increasing importance of the cation-π stabilization as a function of the rising cation size. Alkaline earth metals like Mg^2+^ and Ca^2+^ help produce more stable configurations, as the cations also favor the position over the pyrrole ring (e.g. **1S-Mg-2** and **1S-Ca-2**). Nevertheless, the most stable ones (e.g. **1S-Mg-1** and **1S-Ca-1**), analogue to **1S-Li-2**, feature the SO_2_-π interaction rather than the cation-π interaction.

For multiple-site NHCs (e.g. **3**, ESI Fig. S5b) more stable configurations with close energies appear. This is natural because the cation and SO_2_ can both interact strongly with different N atoms. For NHCs with fused rings (e.g. **6**, ESI Fig. S5c), as the second ring also can stabilize the cation or SO_2_, more stable structures also tend to form. In general, SO_2_ does not succeed in breaking the N-Li bond, which is consistent with the previous observations^8,15,23–27^.

Multiple-configuration mixture instead of the only SO_2_-sandwiched structure may be the true products. This may help us understand the measured spectra better. Benzene imidazolide, a well-studied anionic NHC^7^, serves as a good example. Five stable configurations were predicted when it interacts with SO_2_ and Li^+^ (ESI Figs S4e and S5c). Their diverse S-O bond vibration frequencies are listed in Table 1. Geometries **6S-Li-1** and **6S-Li-2** are generally viewed as the SO_2_-sandwiched near-planar chemisorption product, whose S-O bonds have asymmetric and symmetric stretching vibration frequencies of 987 cm^−1^ and 962 cm^−1^ *and* 979 cm^−1^ and 962 cm^−1^, respectively. For the other three configurations (**6S-Li-3**, **6S-Li-4** and **6S-Li-5**), the corresponding frequencies are blue-shifted to 1226 cm^−1^ and 1037 cm^−1^, 1207 cm^−1^ and 998 cm^−1^ *and* 1201 cm^−1^ and 998 cm^−1^.

**Table 1:** Vibration frequencies of S-O bonds in stable configurations of 6-cation-SO_2_.

Configuration		Asymmetric and symmetric stretching (cm^−1^)	
**6S-Li-1**		987	962
**6S-Li-2**		979	962
**6S-Li-3**		1226	1037
**6S-Li-4**		1207	998
**6S-Li-5**		1201	998

When using NHC-functionalized ionic liquids to capture SO_2_, the presence of a new IR band right below 1000 cm^−1^ (948 cm^−1^ in most reports) is an evidence that SO_2_ is absorbed through chemical interaction, while the presence of other two new bands at about 1324 cm^−1^ and 1143 cm^−1^ implies physical interaction^6,7,10,14,28,31,32^, which is in good agreement with our findings. Therefore, new IR bands near 1000–1300 cm^−1^ after the reaction can indicate whether the SO_2_ absorption product is a mixture of multiple configurations or just the previously focused “sandwiched” structure.

### Li^+^-assisted NHC-SO_2_ interaction

**Figure 6:**
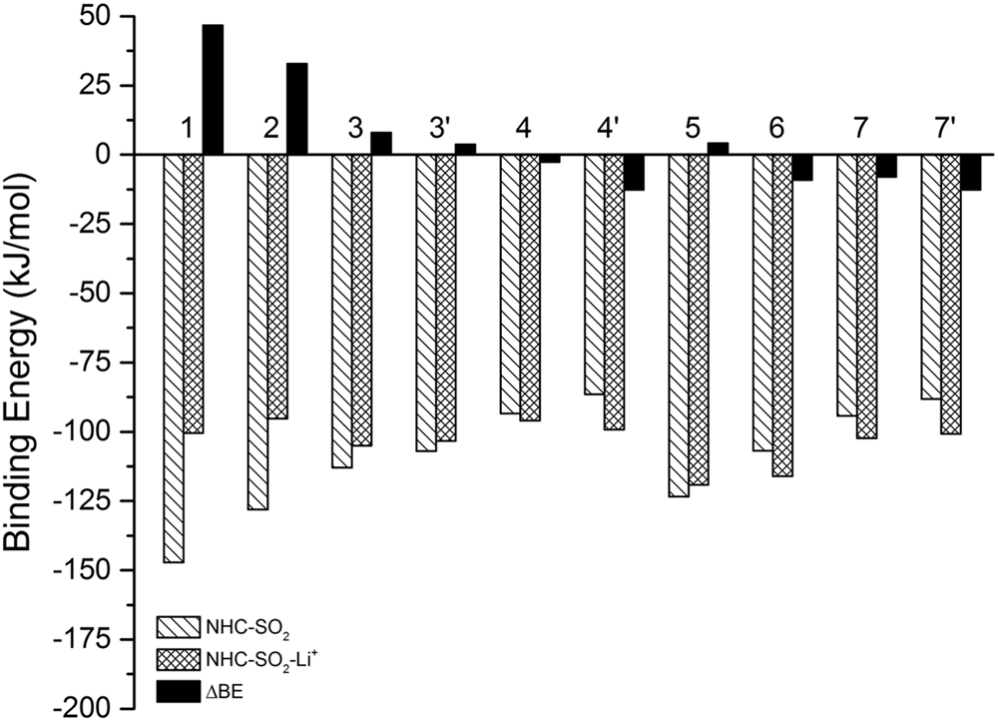
Binding energies between SO_2_ and NHCs alone or with Li^+^. The binding energy differences are represented by filled black bars. Negative ΔBE means that the NHC-SO_2_ interaction is strengthened by cation. Only the most stable configurations are used. For **3**, **4** and **7**, the reaction sites are on the N atom labelled as 1 in Fig. 1. For **3′**, **4′** and **7′**, the sites are at the N2 site.

In Fig. 6, we present the SO_2_ binding energy change between the [NHC-Li^+^] complexes and the NHCs. Overall, the introduction of Li^+^ cation does not exhibit obvious influences (usually |ΔBE| < 20 kJ mol^−1^). Notably, for single-ringed **1**–**3** and double-ringed **5**, the presence of Li^+^ even has a slight to moderate suppressing effect, agreeing with the previous reports^8,23,24^. It is also crucial to highlight that with the increasing number of nitrogen atoms in both single-ringed and double-ringed structure, the difference in NHC-SO_2_ binding energy with or without Li^+ ^decreases gradually. Although cation affects the structural properties of the NHC-SO_2_ adducts substantially, it only has a slight influence on the binding energies.

### Other metal cation-assisted NHC-SO_2_ interactions

**Figure 7:**
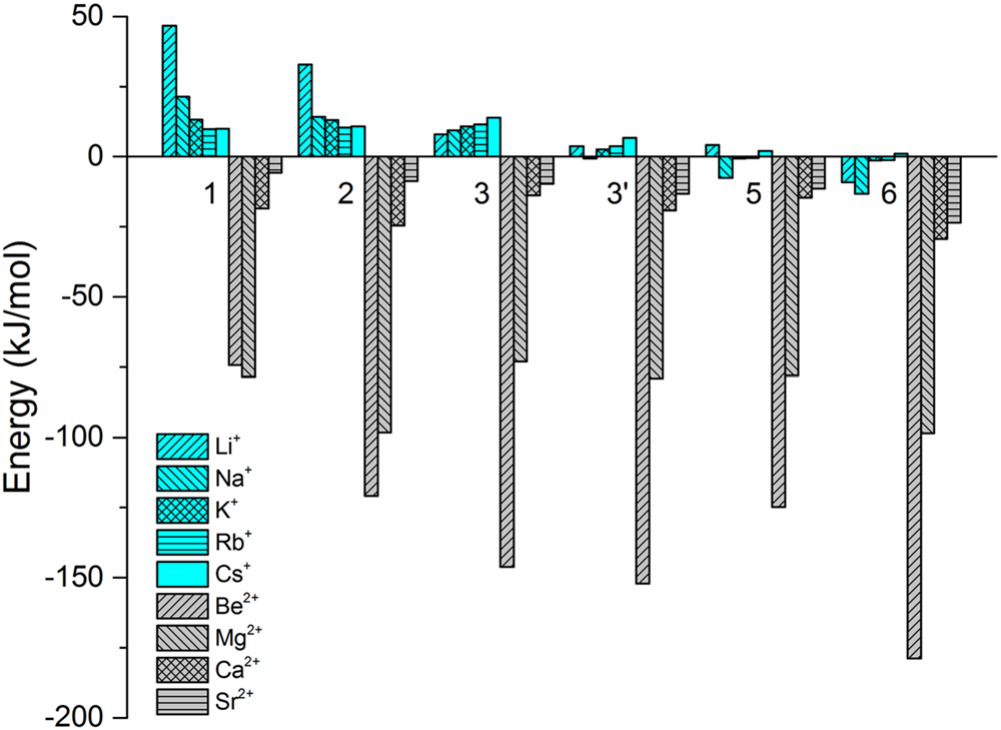
Binding energy differences (ΔBE) of SO_2_ to selected NHCs in the presence of monovalent (bright blue) and divalent cations (gray).

NHC-SO_2_ interactions with other alkali and alkaline earth metals were also investigated. Most monovalent cations, *i*.*e*., alkali metal ions have very limited (weakening) effects on the interaction of NHC-SO_2_ complex, while all the bindings are enhanced by divalent cations, especially by the small ones like Be^2+^ and Mg^2+^(|BE| > 100 kJ mol^−1^, Fig. 7 and ESI Fig. S6), whose smaller size and higher charge are critical.

### Correlation between structure and binding energy

From the above analysis we know there is a big variation in the most stable configurations of NHC-cation-SO_2_ (Fig. 5 and ESI Figs S4–5). Particularly, among some of the most stable configurations, the N atoms are not necessarily the only interacting site and the NHC ring sometimes interact with the cation and SO_2_ as a whole. Thus, a simple descriptor such as NBO nitrogen lone pair orbital energy, used in the case of CO_2_^22^, fails to correctly describe the binding energy, and so does the NBO charge unsurprisingly (ESI Fig. S7). In other words, there still lacks a simple and accurate descriptor to link the reactivity of NHC-cation and SO_2_.

### Comparison between cation-assisted NHC-SO_2_ and NHC-CO_2_ interactions

In the previously studied NHC-cation-CO_2_ system^22^, only the *planar* NHC-cation was identified as the most stable configuration (e.g. ESI, Fig. S1a, pyrrolide-Li^+^**(1 + Li)**, Φ_LiNCH_ = 1.0°). However, this simple and uniform picture is now found to be incomplete: the most stable configuration of the NHC-cation pair is NHC-dependent. For example, for pyrrolide and 1,2,3-triazolide, their most stable complexes with Li^+^ assume very different geometries (ESI Fig. S1). The planar **1 + Li** is only meta-stable and the (*η*^5^-NHC)Li configuration is more stable by 45 kJ mol^−1^, while for **3**(1,2,3-triazolide) + Li, the order of configuration stability is reversed (ESI Fig. S1 and the accompanying text). Yet the most stable configuration of the final NHC-cation-CO_2_ product remains planar (ESI Fig. S5a). Thus, the cation effect should also be NHC-dependent. Moreover, although the whole observation applies to Be^2+^, more study is definitely needed to map out the detailed relationship between other cations and NHCs.

When the cation effects in both anion-CO_2_ and anion-SO_2_ systems are re-evaluated (ESI Fig. S8) using the *newly-identified* most stable NHC-cation configurations, different results are obtained. Unlike the previous study^22^, the introduction of Li^+^ cation does not strengthen the binding between CO_2_ *and* pyrrolide but causes a weak inhibiting effect (0 < ΔBE < 25 kJ mol^−1^, the weakening effect is weaker than in the SO_2_ case). The further study of the interactions between pyrrolide-CO_2_ and other alkali and alkaline earth metals reveals that monovalent cations and even divalent cations also have limited *weakening* effects on the interactions (ESI Fig. S9). This is because the most stable pyrrolide-cation pair configuration has the cation hoving over the NHC ring while the most stable configuration of the final pyrrolide/imidazolide-cation-CO_2_ complexes assume planar structure (ESI Fig. S5a vs. Fig. S1). The shift of the cation’s position costs about more than 50 kJ mol^−1^, rendering the cation a weakening rather than an enhancing role. For other NHCs like imidazolide, the cation effect is more complex and is cation dependent (ESI Fig. S9), which is also a result of the energy difference between the configurations the NHC-cation pair assumes before and after interacting with CO_2_.

Similarly, for NHC-SO_2_ systems, the cation effects are also cation dependent. Monovalent cations usually have small weakening effect and divalent cations mostly show enhancing effect. In contrast to the CO_2_ case, the NHC-cation-SO_2_ complex can assume multiple non-planar forms (particularly for divalent cations, as they prefer to bind to multiple sites in the ring) and the cation’s position over the ring can be more or less kept (Fig. S5b) after the SO_2_ attachment, which reduces the weakening effect of cation substantially. As a result, all bindings of the NHC-SO_2_ complexes are enhanced by divalent cations.

## Conclusions

The cation effects on the interactions between anionic N-containing heterocycles (NHCs) and SO_2_ have been carefully examined using DFT calculations. Cation, along with NHC, makes SO_2_ binding much more complex than its CO_2_ counterpart. Particularly, multiple stable configurations with *close* energies may coexist and their interconversion are possible at ambient conditions, which is crucial to properly understand the SO_2_ capture products. This structural diversity makes the relationship between the structures of NHCs and their reactivity with SO_2 _ much less clear than those observed in the CO_2_ case.

However, monovalent cations (Li^+^, Na^+^, K^+^, Rb^+^, Cs^+^) and big divalent cations (Ca^2+^ and Sr^2+^) usually show very limited influences on the binding energy between SO_2_ and NHC, either weakening or strengthening the binding by less than 20 kJ mol^−1^. Only for pyrrolide and imidazolide, their bindings with SO_2_ are weakened by Li^+^ by over 25 kJ mol^−1^. Small bivalent cations like Be^2+^ and Mg^2+^ promote the binding by over 100 kJ mol^−1^, which indicates the electrostatic nature in NHC-M^2+^-SO_2_ interaction.

In summary, the products of SO_2_ capture using materials consisting of cation-anion pairs are possibly mixture of multiple stable structural isomers, which should be considered when designing new SO_2_ capture materials.

## Computational Methods

The Gaussian 09 package^33^ was chosen to conduct all the calculations. Geometry optimizations were carried out unrestrained at the model chemistry of B3LYP^34^/6–311 ++ G(d, p)^35^ (SDD^36^ for the third to fifth period elements), which has shown appreciable performance over a wide range of NHCs^5,7,8,10,12,24,28^. Frequency analyses with the same model chemistry followed the structure optimization to identify energy minimum.

The binding energy between SO_2_ and NHCs is defined in the following equation:

$${E}_{{\rm{binding}}}={E}_{{\text{sorbent} \mbox{-} \text{SO}}_{2}}-({E}_{{\rm{sorbent}}}+{E}_{{{\rm{SO}}}_{2}})$$

where ${E}_{{\text{sorbent} \mbox{-} \text{SO}}_{2}}$, *E*_sorbent_ and ${E}_{{{\rm{SO}}}_{2}}$ stand for the electronic energies of the complex, the pure sorbent, and SO_2_, respectively. To help understand the NHC-cation-SO_2_ interactions, constrained optimizations were performed by scanning the N-S distance (*d*_N-S_) at 0.05 Å intervals and all the transformations between two stable configurations were achieved using the Intrinsic Reaction Coordinate (IRC) method. The same methods were used for natural resonance theory (NRT)^37–39^, natural bond orbital (NBO) and NBO charge calculations, all in the NBO 6 program^40,41^. In addition, the Molclus program was utilized to search for the multifarious stable configurations of the NHC-cation-SO_2_ complexes^30^.

## Electronic supplementary material

Supporting Information

## References

[1] Ma, X. X., Kaneko, T., Tashimo, T., Yoshida, T. & Kato, K. Use of limestone for SO_2_ removal from flue gas in the semidry FGD process with a powder-particle spouted bed. *Chem. Eng. Sci.* **55**, 4643–4652 (2000).

[2] Gutiérrez Ortiz, F. J., Vidal, F., Ollero, P., Salvador, L. & Cortés, V. Pilot-plant technical assessment of wet flue gas desulfurization using lime stone. *Ind. Eng. Chem. Res.* **45**, 1466–1477 (2006).

[3] Gao X (2010). Gas-liquid absorption reaction between (NH_4_)_2_SO_3_ solution and SO_2_ for ammonia-based wet flue gas desulfurization. Appl. Energ..

[4] Srivastava RK, Jozewicz W (2011). Flue gas desulfurization: the state of the art. J. Air Waste Manage..

[5] Wang CM (2011). Highly efficient and reversible SO_2_ capture by tunable azole-based ionic liquids through multiple-site chemical absorption. J. Am. Chem. Soc..

[6] Yang DZ (2013). Reversible capture of SO_2_ through functionalized ionic liquids. ChemSusChem.

[7] Cui GK (2014). Highly efficient SO_2_ capture by phenyl-containing azole-based ionic liquids through multiple-site interactions. Green Chem..

[8] Cui GK (2015). Acylamido-based anion-functionalized ionic liquids for efficient SO_2_ capture through multiple-site interactions. ACS Sustain. Chem. Eng..

[9] Shannon MS (2015). Chemical and physical absorption of SO_2_ by N-functionalized imidazoles: experimental results and molecular-level insight. Ind. Eng. Chem. Res..

[10] Zhang FT (2016). Improving SO_2_ capture by basic ionic liquids in an acid gas mixture (10% vol SO2) through tethering a formyl group to the anions. RSC Adv..

[11] Rodriguez-Albelo LM (2017). Selective sulfur dioxide adsorption on crystal defect sites on an isoreticular metal organic framework series. Nat. Commun..

[12] Che SY (2017). Designing an anion-functionalized fluorescent ionic liquid as an efficient and reversible turn-off sensor for detecting SO_2_. Chem. Commun..

[13] Bhattacharyya, S. *et al*. Interactions of SO_2_-containing acid gases with ZIF-8: structural changes and mechanistic investigations. *J. Phys. Chem.**C*** 120**, 27221–27229 (2016).

[14] Wang CM (2013). Highly efficient SO_2_ capture through tuning the interaction between anion-functionalized ionic liquids and SO_2_. Chem. Commun..

[15] Zeng SJ (2014). Efficient and reversible capture of SO_2_ by pyridinium-based ionic liquids. Chem. Eng. J..

[16] Garcia G, Atilhan M, Aparicio S (2017). Simultaneous CO_2_ and SO_2_ capture by using ionic liquids: a theoretical approach. Phys. Chem. Chem. Phys..

[17] Yang ZZ, He LN (2014). Efficient CO_2_ capture by tertiary amine-functionalized ionic liquids through Li^+^-stabilized zwitterionic adduct formation. Beilstein J. Org. Chem..

[18] Zhang S (2014). Equimolar carbon absorption by potassium phthalimide and *in situ* catalytic conversion under mild conditions. ChemSusChem.

[19] Poloni R, Smit B, Neaton JB (2012). Ligand-assisted enhancement of CO_2_ capture in metal-organic frameworks. J. Am. Chem. Soc..

[20] Poloni R, Lee K, Berger RF, Smit B, Neaton JB (2014). Understanding trends in CO_2_ adsorption in metal-organic frameworks with open-metal sites. J. Phys. Chem. Lett..

[21] McDonald TM (2015). Cooperative insertion of CO_2_ in diamine-appended metal-organic frameworks. Nature.

[22] Tang HR, Lu DM, Wu C (2015). Cation-assisted interactions between N-heterocycles and CO_2_. Phys. Chem. Chem. Phys..

[23] Cui YH, Chen YF, Deng DS, Ai N, Zhao Y (2014). Difference for the absorption of SO_2_ and CO_2_ on [P_nnnm_][Tetz] (n = 1, m = 2, and 4) ionic liquids: a density functional theory investigation. J. Mol. Liq..

[24] Chen KH (2015). Designing of anion-functionalized ionic liquids for efficient capture of SO_2_ from flue gas. AIChE J..

[25] Li HP (2015). Theoretical evidence of charge transfer interaction between SO_2_ and deep eutectic solvents formed by choline chloride and glycerol. Phys. Chem. Chem. Phys..

[26] Tan K (2015). Competitive coadsorption of CO_2_ with H_2_O, NH_3_, SO_2_, NO, NO_2_, N_2_, O_2_, and CH_4_ in M-MOF-74 (M = Mg, Co, Ni): the role of hydrogen bonding. Chem. Mater..

[27] Zhang XM (2016). Cyano-containing protic ionic liquids for highly selective absorption of SO_2_ from CO_2_: experimental study and theoretical analysis. Ind. Eng. Chem. Res..

[28] Cui GK (2015). Tuning the basicity of cyano-containing ionic liquids to improve SO_2_ capture through cyano-sulfur interactions. Chem. Eur. J..

[29] Lee HM, Youn IS, Saleh M, Lee JW, Kim KS (2015). Interactions of CO_2_ with various functional molecules. Phys. Chem. Chem. Phys..

[30] Lu, T. Molculs program, website: http://www.keinsci.com/research/molclus.html.

[31] Cui GK (2012). Highly efficient SO_2_ capture by dual functionalized ionic liquids through a combination of chemical and physical absorption. Chem. Commun..

[32] Ding F (2014). Highly efficient and reversible SO_2_ capture by surfactant-derived dual functionalized ionic liquids with metal chelate cations. Ind. Eng. Chem. Res..

[33] Frisch, M. J. *et al*. Gaussian 09, Revision B.01. Gaussian, Inc., Wallingford CT (2009).

[34] Becke AD (1993). Density-functional thermochemistry. 3. the role of exact exchange. J. Chem. Phys..

[35] Hehre, W. J., Radom, L., Schleyer, P. V. R. & Pople, J. A. *Ab Initio Molecular Orbital Theory*. Wiley, New York (1986).

[36] Fuentealba P, Preuss H, Stoll H, Szentpaly Lv (1982). A proper account of core-polarization with pseudopotentials: single valence-electron alkali compounds. Chem. Phys. Lett..

[37] Glendening ED, Weinhold F (1998). Natural resonance theory: I. general formalism. J. Comput. Chem..

[38] Glendening ED, Weinhold F (1998). Natural resonance theory: II. natural bond order and valency. J. Comput. Chem..

[39] Glendening ED, Badenhoop JK, Weinhold F (1998). Natural resonance theory: III. chemical applications. J. Comput. Chem..

[40] Glendening, E. D. *et al*. NBO 6.0., Theoretical Chemistry Institute, University of Wisconsin, Madison (2013).

[41] Glendening ED, Landis CR, Weinhold F (2012). Natural bond orbital methods. Wiley Interdiscip. Rev.: Comput. Mol. Sci..

