# Supplementary material for: The role of cations in the interactions between anionic N-heterocycles and SO2
Source: Sci Rep. 2018 May 8;8:7284. doi: 10.1038/s41598-018-25432-6 (PMC5940702; doi:10.1038/s41598-018-25432-6)
Supplement: Supplementary file 1 — Supporting Information [file 41598_2018_25432_MOESM1_ESM.docx]

**The role of cations in the interactions between anionic N-heterocycles and SO_2_**

Chenchen Li^1^, Dongmei Lu^2^, and Chao Wu^1,*^

^1^Frontier Institute of Science and Technology, Xi'an Jiaotong University, Xi'an, Shaanxi 710054, China.

^2^Department of Chemistry, School of Science, Xi'an Jiaotong University, Xi'an, Shaanxi 710049, China.

*[corresponding.chaowu@mail.xjtu.edu.cn](mailto:corresponding.chaowu@mail.xjtu.edu.cn)

**Supporting Information**

**Contents**

Figure S1 Stable configurations of NHC-cation pairs……………………………………………...2

Figure S2 Two metastable configurations and one stable configuration of the pyrrolide-Li^+^-CO_2_ complex *and* their interconversion……………………………………………………………….....3

Figure S3 NBO and NRT analyses of pyrrolide-Li^+^-SO_2_…………………………………………..4

Figure S4 Stable structures of other NHC-Li^+^-SO_2_ complexes identified by the PES………….….6

Figure S5 Stable configurations of three selected NHC-cation-CO_2_/SO_2_ complexes……………...7

Figure S6 Binding energies between SO_2_ and NHCs alone or with Ca^2+^…………………………..8

Figure S7 Binding energy of single-ringed NHCs and SO_2_ with Li^+^ versus the NBO orbital energy of the lone pair of the reacting N atom and the NBO charge of the reacting N atom……………....9

Figure S8 Binding energies between CO_2_/SO_2_ and selected NHCs alone or with Li^+^…………....10

Figure S9 Binding energy differences of CO_2_/SO_2_ to selected NHCs in the presence of monovalent and divalent cations .............................................................................................………………...10

Reference………………………………………………………………………………………….10

**Stable configurations of NHC-cation pairs**

Multiple stable configurations with a large energy difference were also observed in NHC-cation pair. As mentioned earlier (Fig. 2a), pyrrolide-Li^+^ actually has at least two different conformations, which are named as **1+Li** and **1+Li-1** (Fig. S1a). In our previous work, we only identified the planar ion pair **1+Li** (Φ_LiNCH_ = 1.0 °) as the most stable one, a derived result of neutral NHC (pyridine) and Li^+^.^[1]^ In this work, we found that **1+Li-1** ((*η*^5^-NHC)Li, the Li-N and four Li-C bond lengths are 2.01 Å, 2.06 Å, 2.06 Å, 2.18 Å and 2.18 Å, respectively) is more stable than **1+Li** by 45 kJ mol^-1^. Transformation between these two pyrrolide-Li pairs and transition state **1+Li-TS** only with interaction between the N atom and Li atom are shown in black dots in Fig. S1a. However, for multiple-site anions, e.g. 1,2,3-triazolide (**3**, shown in red dots in Fig. S1a), the planar bidentate adduct **3+Li** is 46 kJ mol^-1^ lower than the (*η*^5^-**3**)Li **3+Li-1**. This process requires a little bigger barrier and the transition state **3+Li-TS** is nearly a perpendicular one (Φ_LiNNN_ = 92.2 °). Bivalent cation like Be^2+^, in addition, was also studied, which shows consistent result with the case of monovalent cation (Fig. S1b).


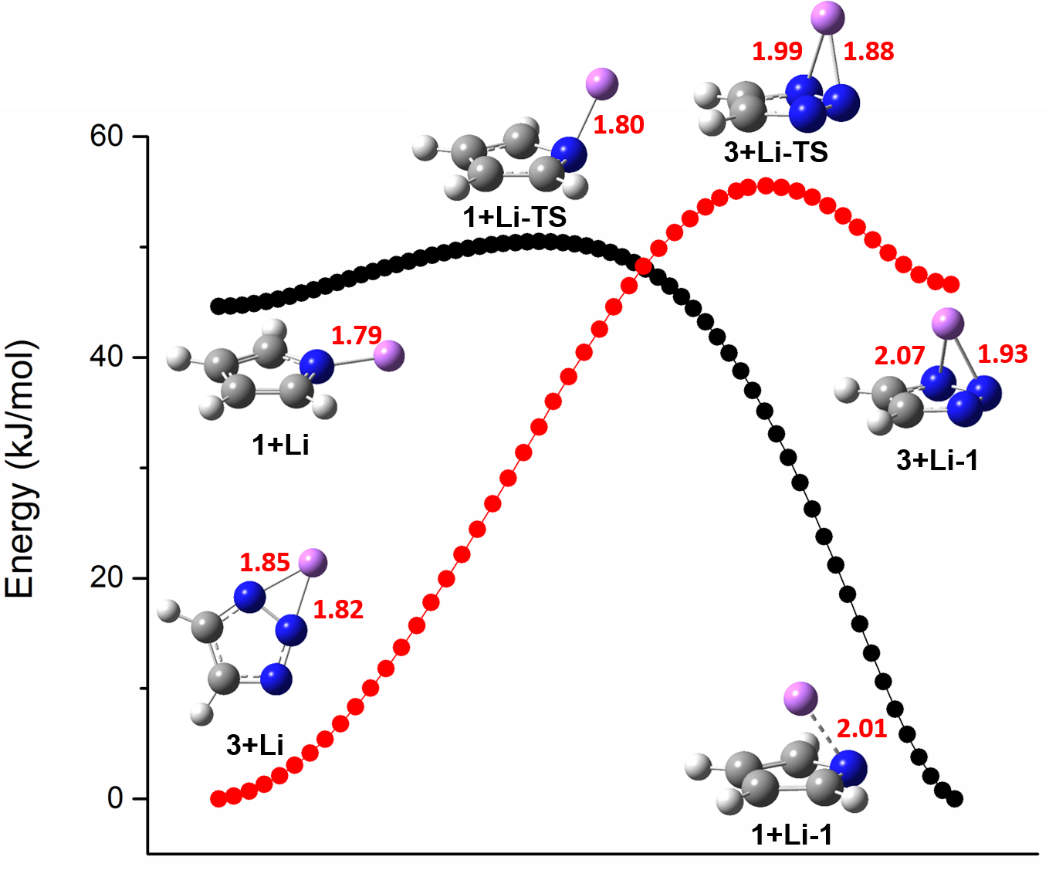


(a)


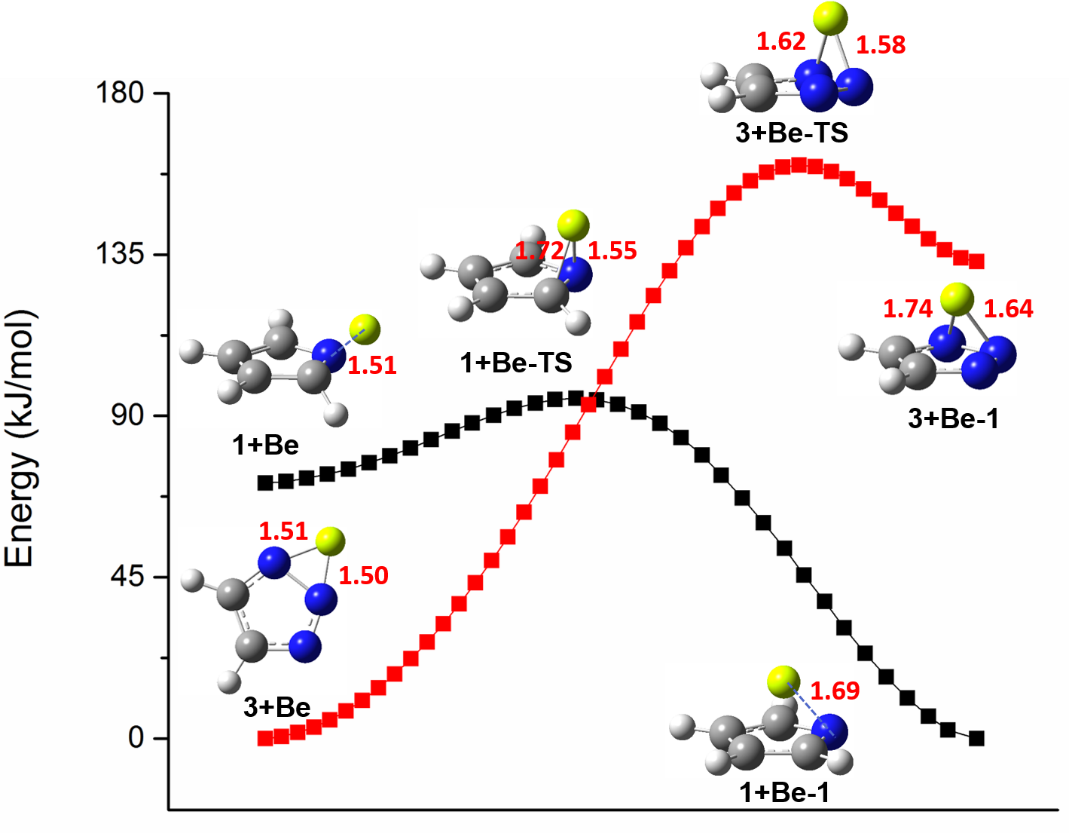


(b)

Figure S1. Two stable configurations of (a) **1+Li** (black dots) and **3+Li** (red dots) complexes with their interconversions *and* (b) **1+Be** (black squares) and **3+Be** (red squares) complexes with their interconversions.


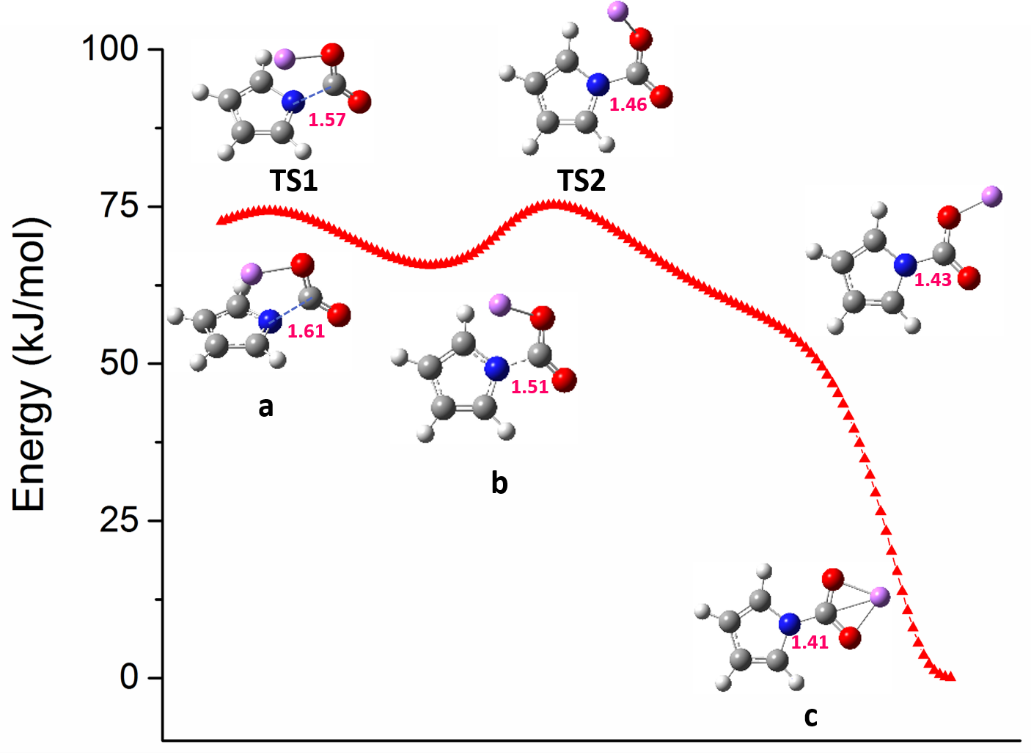


Figure S2. Two metastable configurations (**a** and **b**) of the **1**-Li^+^-CO_2_ complex identified by the PES *and* the conversion to the most stable configuration **c**. N-C bond lengths (Å) are labelled.


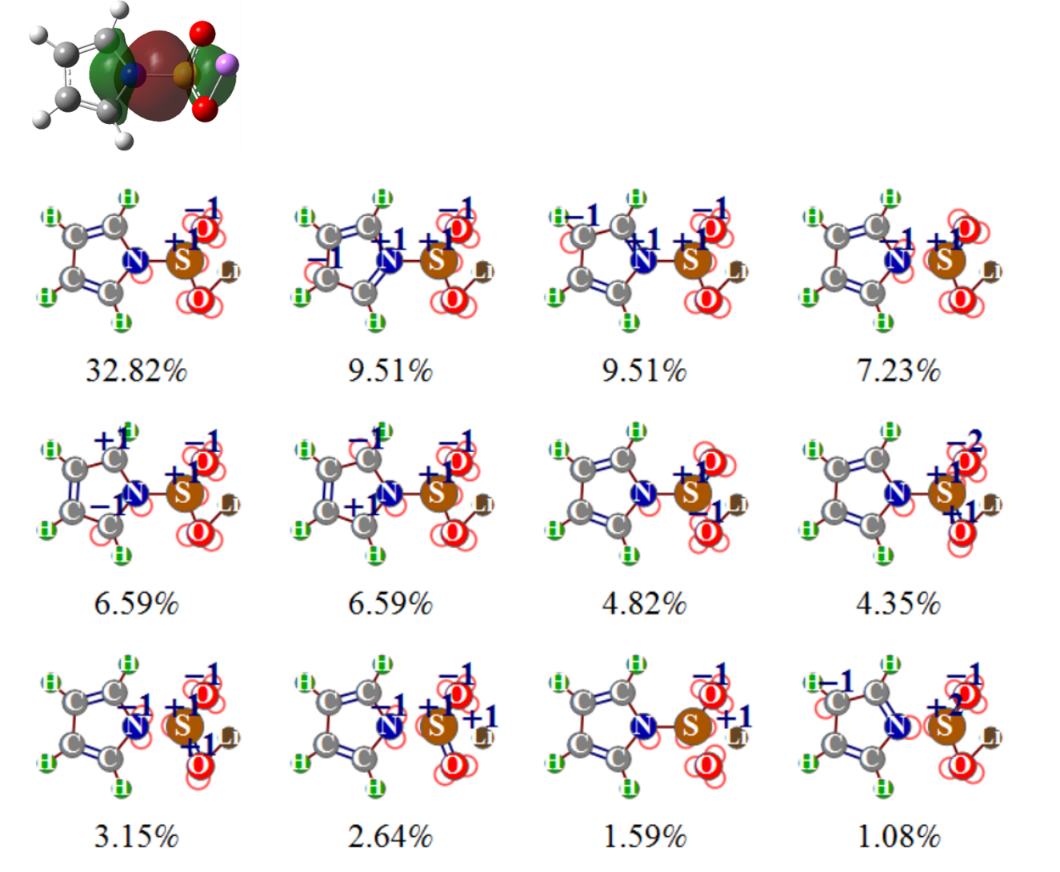


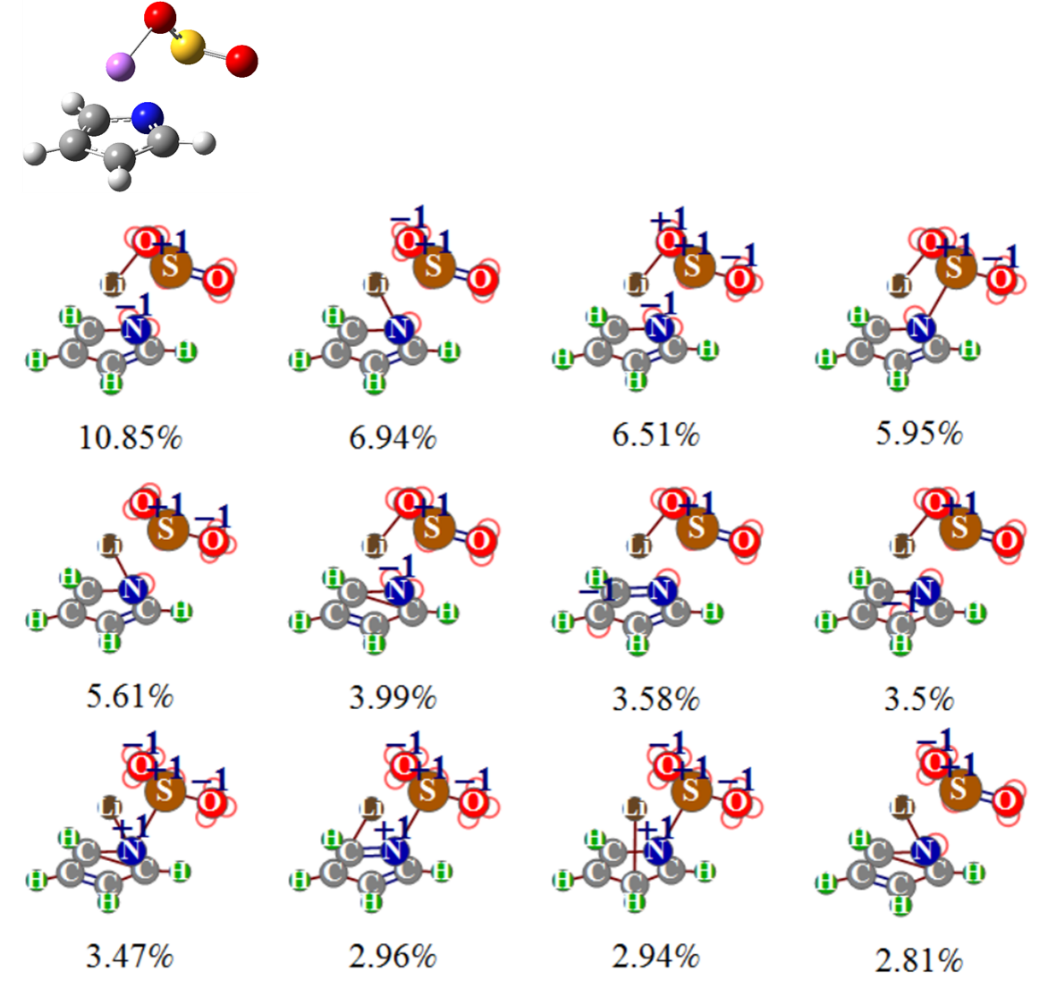


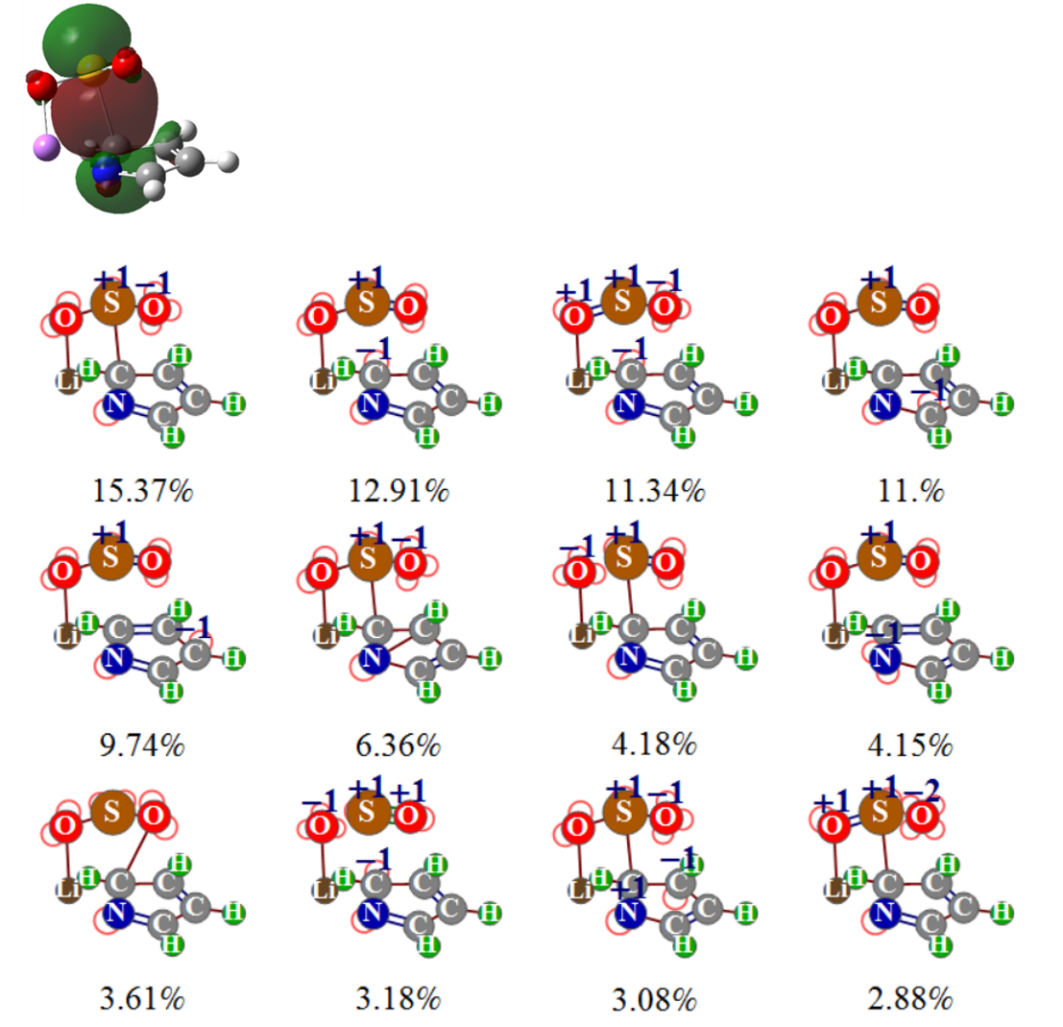


Figure S3. NBO and NRT analyses of three different configurations of (pyrrolide-Li^+^-SO_2_) complexes. Each half circle represents a pair of electrons. Formal charges are placed above the corresponding atoms.


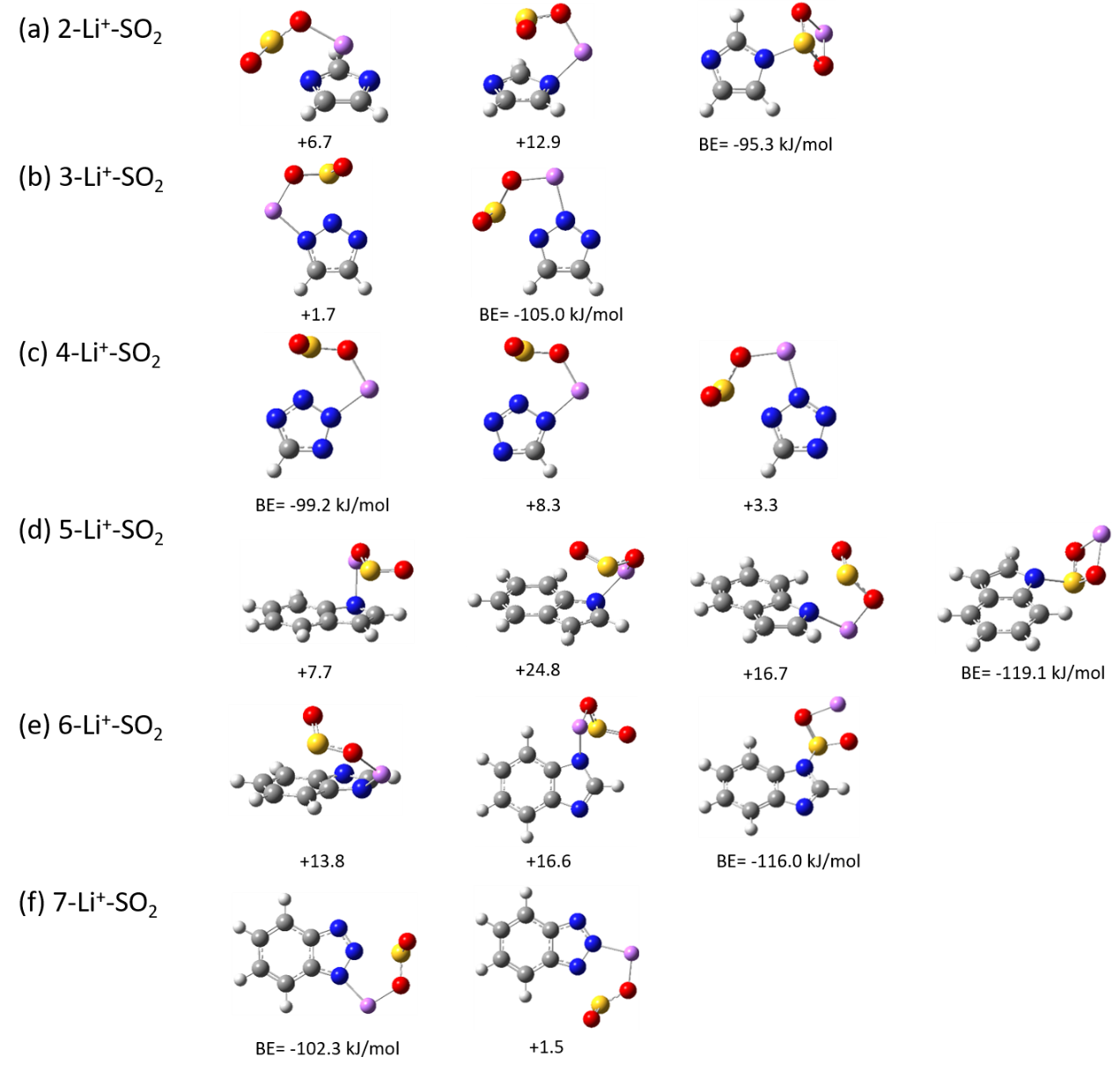


Figure S4. Stable structures of (a) **2**-Li^+^-SO_2_, (b) **3**-Li^+^-SO_2_, (c) **4**-Li^+^-SO_2_, (d) **5**-Li^+^-SO_2_, (e) **6**-Li^+^-SO_2_ and (f) **7**-Li^+^-SO_2_ complexes identified by the PES. The binding energy or the relative binding energy with respect to the most stable one (in kJ mol^-1^) are given below each configuration.


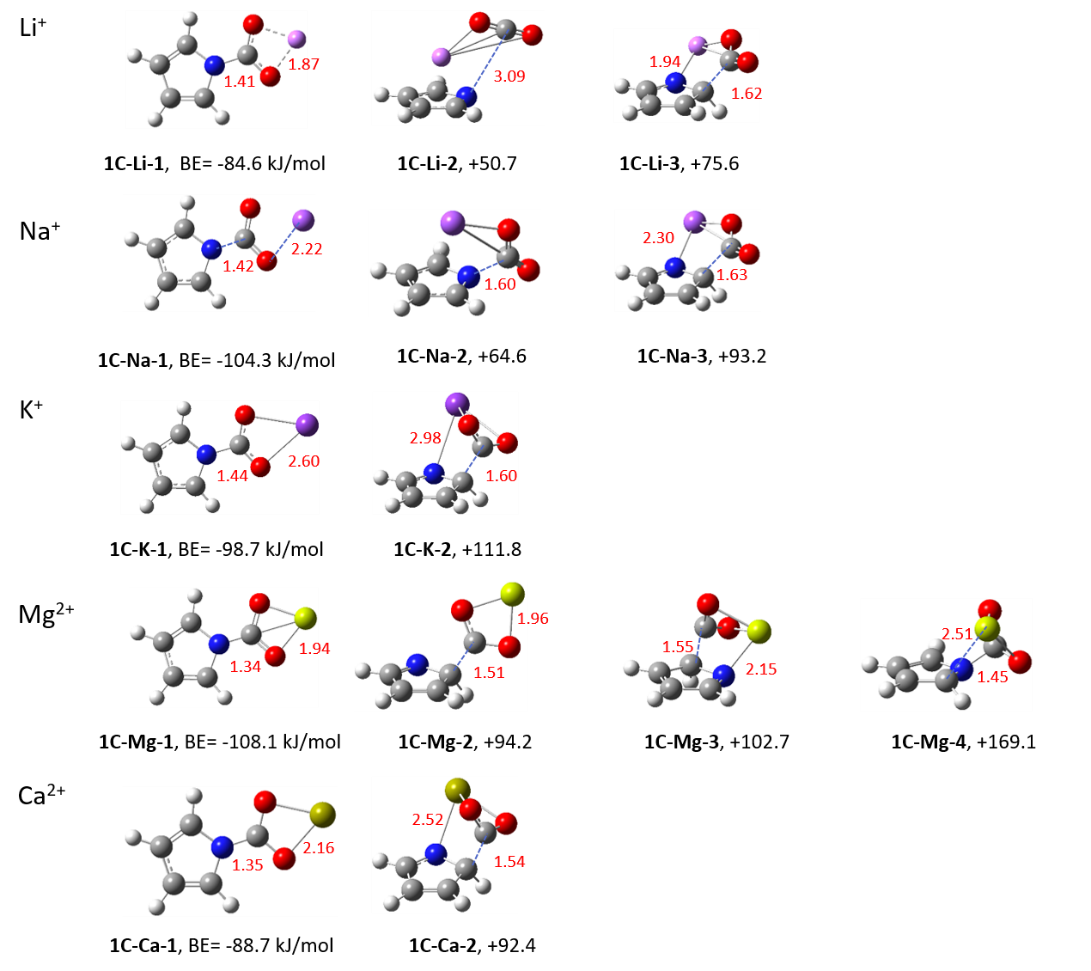


(a)


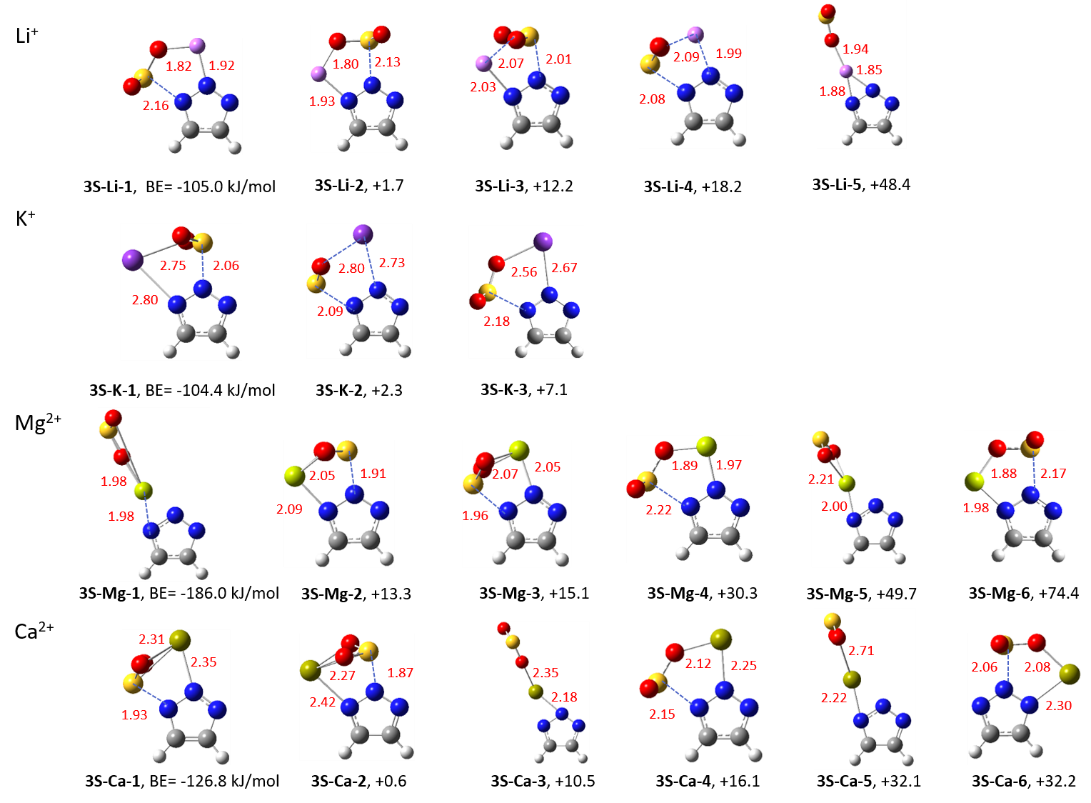


(b)


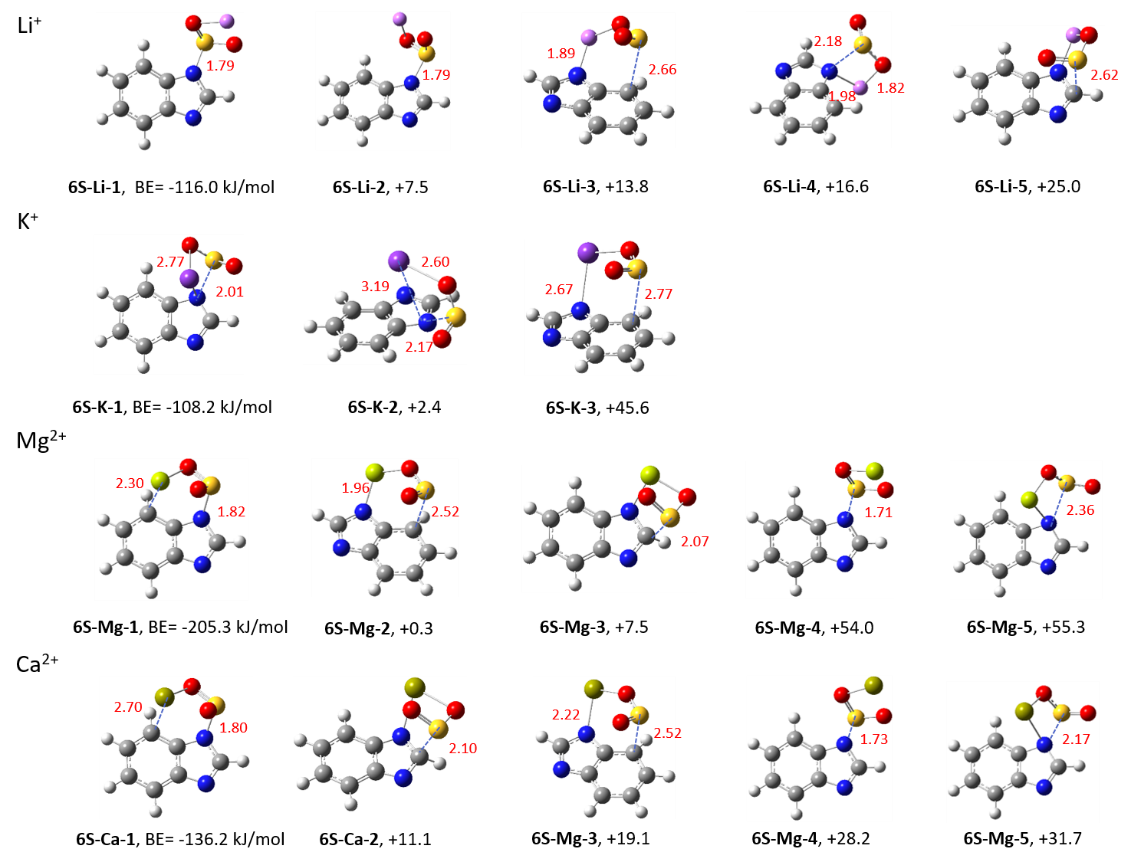


(c)

Figure S5. Stable configurations of (a) **1**-cation-CO_2_, (b) **3**-cation-SO_2_ and (c) **6**-cation-SO_2_ complexes. The name and the binding energy (or the relative binding energy with respect to the most stable one, in kJ mol^-1^) are given below each configuration. Key bond lengths (Å) are labelled to help understand the structures better.


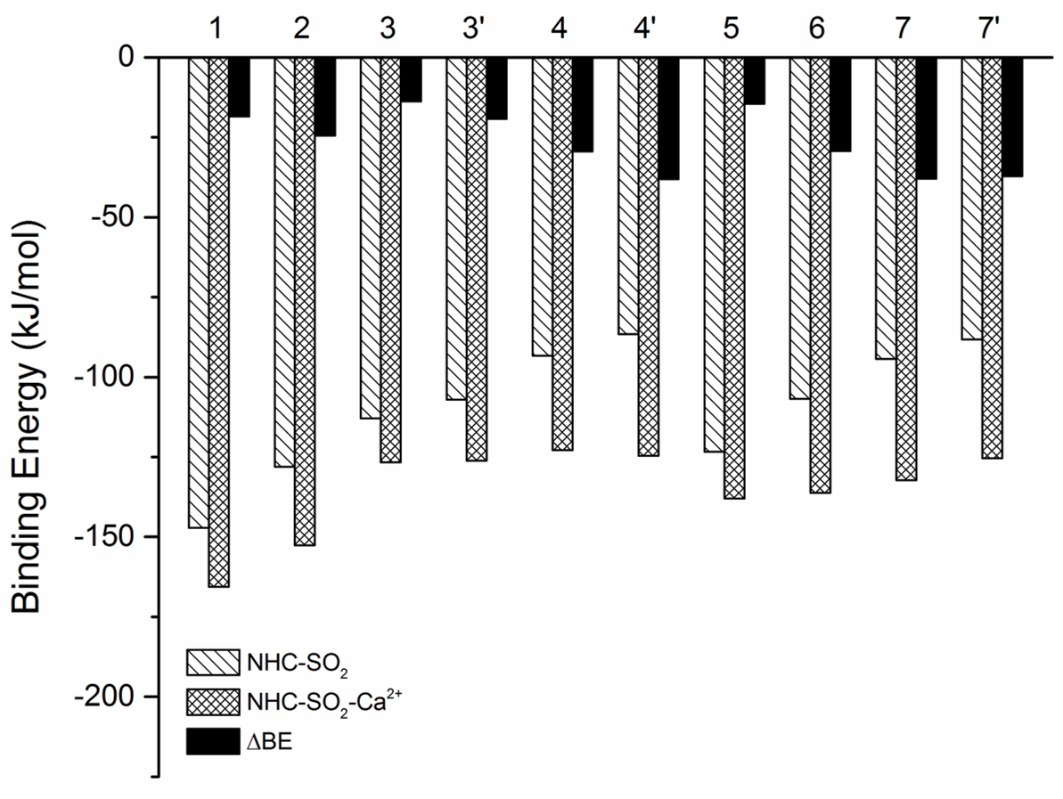


Figure S6. Binding energies between SO_2_ and NHCs alone or with Ca^2+^.


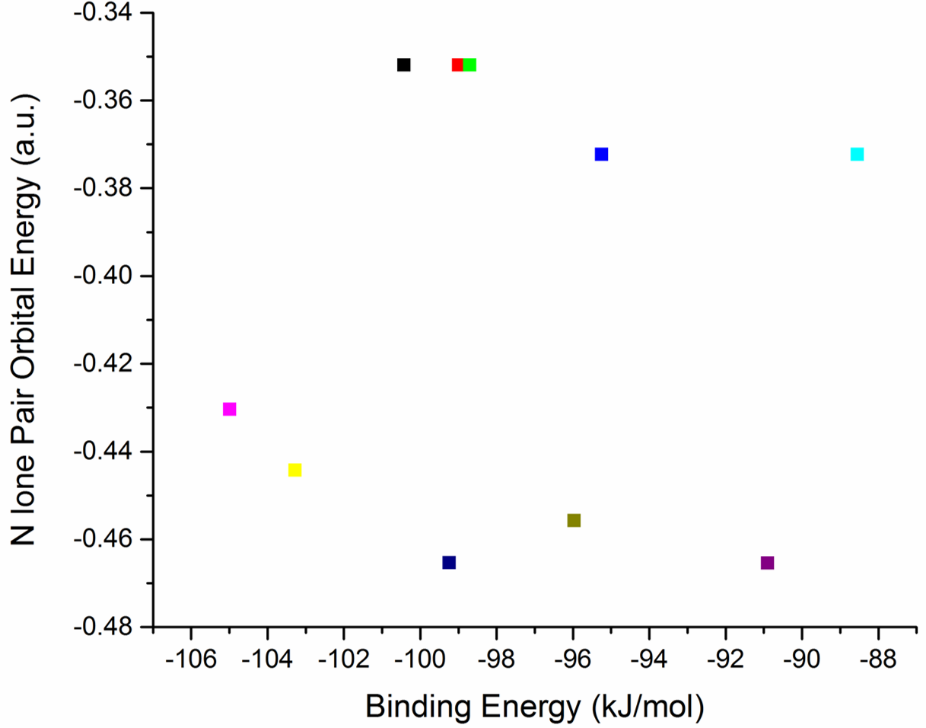


(a)


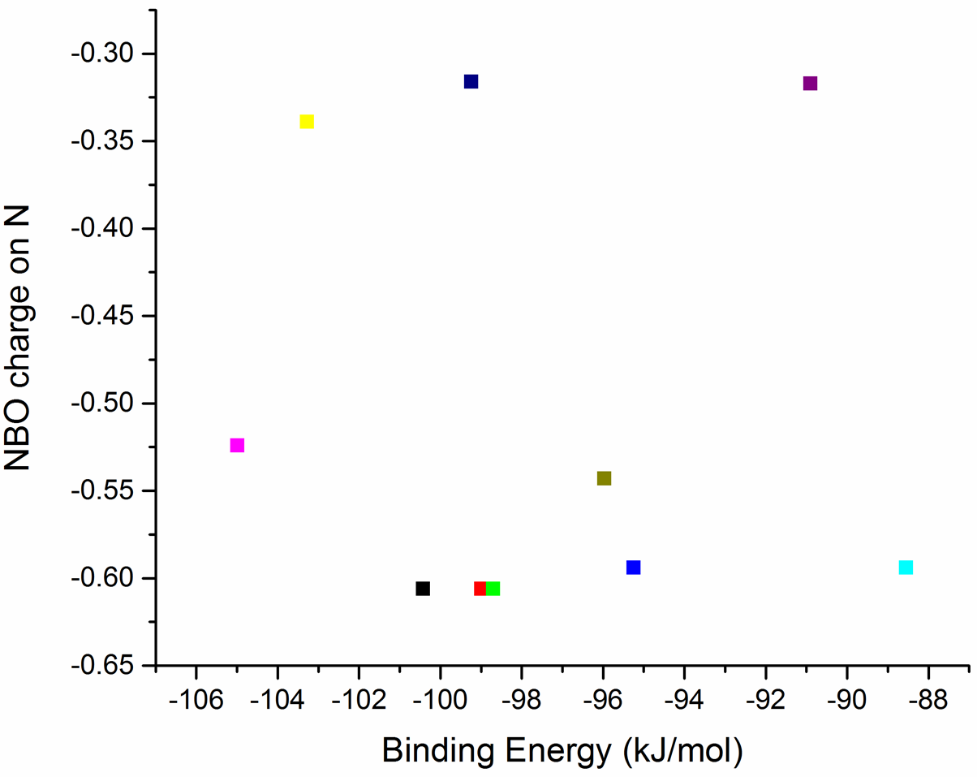


(b)

Figure S7. Binding energy of anionic single-ringed NHCs and SO_2_ with Li^+^ versus (a) the NBO orbital energy of the lone pair of the reacting N atom *and* (b) the NBO charge of the reacting N atom.


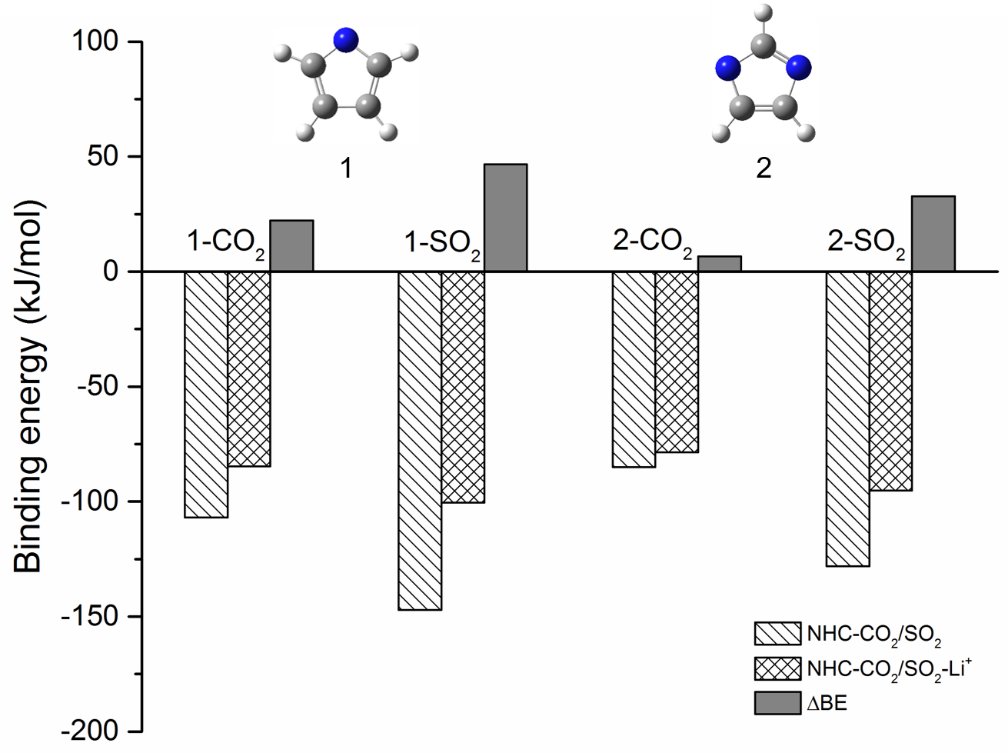


Figure S8. Binding energies between CO_2_/SO_2_ and the selected NHCs alone or with Li^+^. The binding energy differences are represented by the filled gray bars. Positive ΔBE means that the NHC-CO_2_/SO_2_ interaction is weakened by cation. Only the most stable configurations of the complexes are used. Insets: the configurations of pyrrolide (**1**) and imidazolide (**2**).


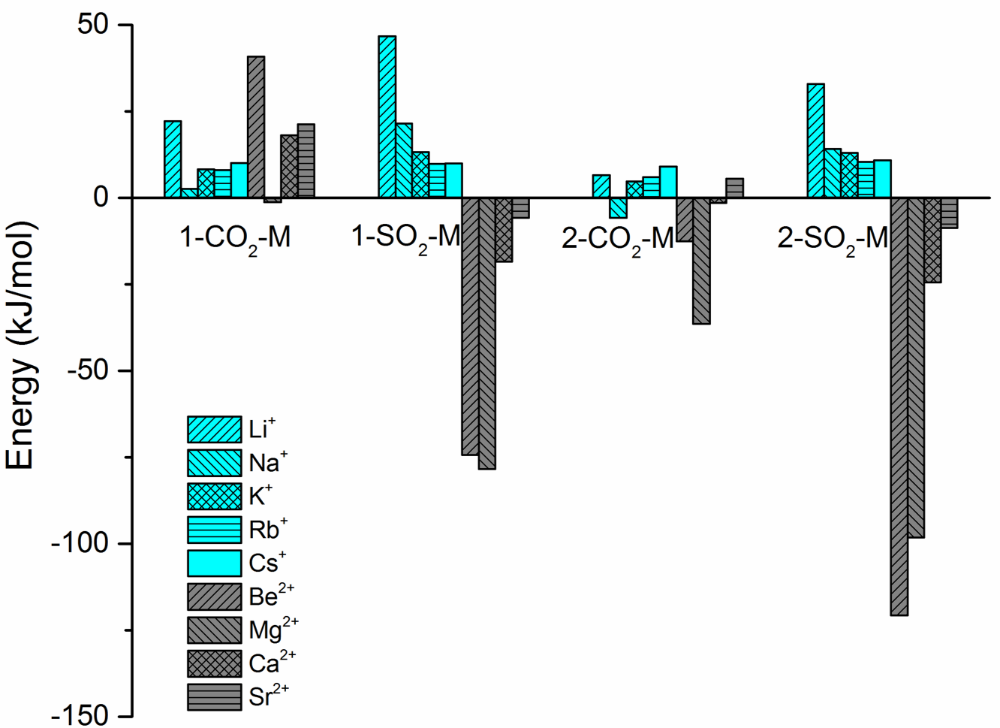


Figure S9. Binding energy differences (ΔBE) of CO_2_/SO_2_ to the selected NHCs in the presence of monovalent (bright blue) and divalent cations (gray). Labels for 1 and 2 see Figure S8.

**Reference**

1 Tang, H. R., Lu, D. M. & Wu, C. Cation-assisted interactions between N-heterocycles and CO_2_. *Phys. Chem. Chem. Phys.* **17**, 15725-15731 (2015).
